# Fatal Systemic Morbillivirus Infection in Bottlenose Dolphin, Canary Islands, Spain

**DOI:** 10.3201/eid2002.131463

**Published:** 2014-02

**Authors:** Eva Sierra, Daniele Zucca, Manuel Arbelo, Natalia García-Álvarez, Marisa Andrada, Soraya Déniz, Antonio Fernández

**Affiliations:** University of Las Palmas de Gran Canaria, Arucas, Canary Islands, Spain

**Keywords:** morbillivirus, bottlenose dolphin, Canary Islands, Spain, viruses, cetacean morbilliviruses

## Abstract

A systemic morbillivirus infection was diagnosed postmortem in a juvenile bottlenose dolphin stranded in the eastern North Atlantic Ocean in 2005. Sequence analysis of a conserved fragment of the morbillivirus phosphoprotein gene indicated that the virus is closely related to dolphin morbillivirus recently reported in striped dolphins in the Mediterranean Sea.

Since the first morbillivirus outbreak affecting bottlenose dolphins (*Tursiops truncatus*) along the mid-Atlantic Coast of the United States during the late 1980s (*1*), some mass die-off episodes affecting the global cetacean population have occurred (*2*–*5*). Three cetacean morbilliviruses have been identified: porpoise morbillivirus, isolated from harbor porpoises that died along the coast of Ireland (*6*); dolphin morbillivirus (DMV), first identified in striped dolphins from the Mediterranean Sea (*2*); and pilot whale morbillivirus, isolated from a long-finned pilot whale stranded in New Jersey, USA (*7*). Other members of the *Paramyxoviridae* family that have affected various marine mammal populations worldwide are phocine distemper virus and canine distemper virus (CDV) (*8*). Since early July 2013, a widespread die-off of >500 bottlenose dolphins occurred along the US mid-Atlantic Coast that probably resulted from morbillivirus infection (*9*).

We report a unique morbillivirus that caused documented systemic infection in a bottlenose dolphin from the eastern North Atlantic Ocean. Although this case occurred nearly 9 years ago, the nucleotide sequence of a fragment of the morbillivirus phosphoprotein (*P*) gene shows high homology with sequences recently reported for morbilliviruses from striped dolphins (*Stenella coeruleoalba*) in the Mediterranean Sea. This information is of value because no other sequences of morbillivirus from bottlenose dolphins are available in GenBank, despite the seriousness of this fatal disease, which causes mass deaths of marine mammals worldwide.

## Case Report

On July 18, 2005, a juvenile female bottlenose dolphin was stranded alive in Arrieta, Lanzarote (Canary Islands, Spain). The animal was 250 cm long and in moderate body condition, according to anatomic parameters. The animal died shortly after stranding, and a complete standardized necropsy was performed within 6 hours after death. Required permission for the management of stranded cetaceans in the Canarian archipelago is issued by the environmental department of the Canary Islands’ government.

Tissues of all the major organs and lesions were collected and stored in neutral buffered 10% formalin fixative solution for histologic and immunohistochemical analyses. Fixed tissue samples were trimmed and then routinely processed, embedded in paraffin, sectioned at 5 μm, and stained with hematoxylin and eosin for examination by light microscopy. A monoclonal antibody against the nucleoprotein of CDV (MoAb CDV-NP, VMRD, Inc., Pullman, WA, USA) known to react with DMV was used as primary antiserum. Immunohistochemical analysis was performed on selected samples of brain, intestinal, lymphatic, pancreatic, pulmonary, renal, and splenic tissues, as described (*4*). Samples of lung, spleen, and brain were collected and held frozen at −80°C until processed for molecular virology.

On gross examination, the most remarkable findings were moderate to severe multiorgan parasitic infection, mainly cirripedes (*Xenobalanus* sp.) in the caudal fin; larval cestodes (*Phyllobothrium* sp. and *Monorygma grimaldi*) within the subcutaneous and peritoneum tissues; nematodes (*Anisakis* spp.), trematodes (*Pholeter* sp.), and taeniform cestodes in the stomachs and intestine, and trematodes (*Nasitrema* sp.) and nematodes (*Crassicauda* sp.) within both pterygoid sinuses; bilateral serous-suppurative and proliferative arthritis at the scapula-humeral junction; and generalized lymphadenomegalia. Within the brain; the leptomeninges were enlarged and fibrotic at the cortex area.

**Figure 1:**
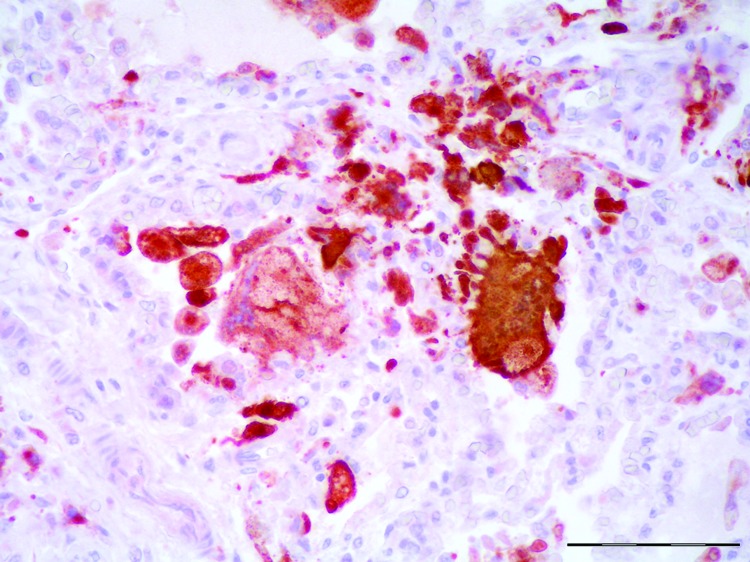
Lung from necropsy of a bottlenose dolphin (*Tursiops truncatus*) that had fatal systemic morbillivirus infection, Canary Islands, Spain, 2005. Positive intracytoplasmic and intranuclear immunoperoxidase staining of morbilliviral antigen (red) within hyperplastic type II pneumocytes, macrophages and multinucleated syncytial cells. Avidin-biotin-peroxidase with Harris hematoxylin counterstain. Scale bar indicates 100 μm.

Histologically, the lesions were consistent with findings from the gross examination. Other histopathologic findings were multifocal nonsuppurative hepatitis, adrenalitis, and bronchointerstitial pneumonia. In lung, a multifocal suppurative bronchitis associated with larval and adult nematode parasites (morphologically identified as *Halocercus* spp. and *Stenurus* spp.) were observed. Alveolar septa were expanded by macrophages and lymphocytes admixed with scattered karyorrhectic debris. Alveoli were often lined by hyperplastic type II pneumocytes and filled with large numbers of multinucleated syncytial cells. The immunohistochemical study demonstrated morbilliviral antigen in bronchiolar epithelium, type 2 pneumocytes, and multinucleate (syncytial) cells from lung (Figure 1).

The syncytia also were found within the lymph nodes, pancreas, spleen, kidney, and intestine, and they were characterized by a moderate amount of eosinophilic cytoplasm and 2 to >20 nuclei, often containing weak stained eosinophilic inclusion bodies. Lymphocytolysis was remarkable within the spleen and multiple lymph nodes and were characterized by loss of cellular detail and accumulation of karyorrhectic and eosinophilic cellular debris. Syncytia from lymph nodes, spleen, pancreas, and kidney also were immunostained against CDV antibody. Severe nonsuppurative meningitis (with >20 layers of lymphohistiocytic cells), perineuritis, and encephalomyelitis were found within the nervous system. Multiple microhemorrhages were a common associated lesion. The brain showed severe inflammatory changes, but only a few neurons were immunopositive, and they were limited to some lymphohistiocytic cells surrounding vessels, glial, and endothelial cells. The epithelial tropism of the virus was demonstrated immunohistochemically within the lung, intestine, kidney, and pancreatic duct epithelium.

Molecular detection of cetacean morbillivirus was performed by a 1-step reverse transcription PCR of a 426-bp conserved region of the *P* gene, as described (*10*). All the samples tested from this dolphin were reverse transcription PCR positive for morbillivirus. Sequences were the same in all positive samples from the animal. We conducted a BLAST (www.ncbi.nlm.nih.gov/blast/Blast.cgi) search to compare sequenced products with sequences described in GenBank for morbillivirus. The common sequence of 411-nt fragment of the *P* gene showed a 99% homology with those sequences obtained in 2007 and 2011 from striped dolphins stranded along the coastline of the Mediterranean Sea.

**Figure 2:**
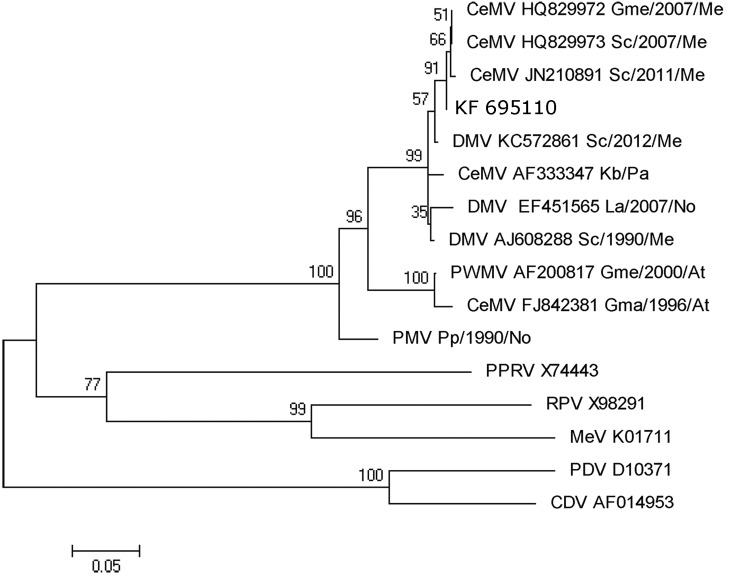
Phylogram of morbillivirus phosphoprotein gene sequences. MEGA 5.0 software (www.megasoftware.net) was used to construct the maximum-likelihood phylogenetic trees. A Tamura-Nei substitution model and a bootstrap resampling (1,000 replicates) were used to assess the reliability of the trees. Bootstrapping values are indicated as percentages next to bifurcations. The new isolate from this study has the GenBank accession no. KF695110. Sequence names include the virus name (CeMV, cetacean morbillivirus; DMV, dolphin morbillivirus; PWMV, pilot whale morbillivirus; PMV, porpoise morbillivirus; PPRV, peste-des-petits-ruminants virus; RPV, rinderpest virus; MeV, measles virus; PDV, phocine distemper virus; CDV, canine distemper virus; GeneBank accession numbers (when available); cetaceans species (Gme, *Globicephala melas*; Sc, *Stenella coeruleoalba*; Kb, *Kogia breviceps*; La, *Lagenorhynchus albirostris*; Gma, *Globicephala macrorhynchus*; Pp, *Phocoena phocoena*; Tt, *Tursiops truncatus);* and the year and the geographic area of the stranding (Me, Mediterranean Sea; Pa, Pacific Ocean; No, North Sea; At, Atlantic Ocean. Scale bar indicates nucleotide substitutions per site.

We used MEGA 5.0 software (www.megasoftware.net) to construct maximum-likelihood phylogenetic trees. A bootstrap resampling (1,000 replicates) was used to assess the reliability of the trees (Figure 2).

## Conclusions

We describe a systemic morbillivirus infection in a bottlenose dolphin in the Canary Islands. A previous morbillivirus infection had been described in a bottlenose dolphin found dead along the Atlantic coast of Mauritania in 1998; the virus was exclusively detected by hybridization in the stomach tissue sample (*11*).

Other cases that illustrate the systemic and neurologic features of the disease have been described in bottlenose dolphins in the Mediterranean Sea (*12*–*14*), and the first reported case of a fatal morbillivirus infection in cetaceans within the Southern Hemisphere (southwestern Pacific Ocean) has been described (*15*). No sequences from these studies were provided to GenBank, although the phylogenetic analyses showed high homology with other DMV. The *P* gene fragment of the virus obtained from the current study is molecularly almost identical to that reported in striped dolphins in the Mediterranean Sea during the last 5 years. This fact supports the hypothesis that transmission occurs between species, as it was reported in the 2006–2007 Mediterranean epizootic between pilot whales (*Globicephala melas* and *G. macrorhynchus*) and striped dolphins (*3*), and demonstrates that dolphin populations of the Mediterranean Sea and the Atlantic Ocean are in contact through the Gibraltar Straits. This particular point is critical to better understand the epidemiology and transmission of morbilliviruses between cetaceans and could be essential to clarifying the infection source of the die-off of bottlenose dolphins alson the East Coast of the United States (*9*).
